# Small sample properties of rare variant analysis methods

**DOI:** 10.1186/1753-6561-8-S1-S13

**Published:** 2014-06-17

**Authors:** Michael D Swartz, Taebeom Kim, Jiangong Niu, Robert K Yu, Sanjay Shete, Iuliana Ionita-Laza

**Affiliations:** 1 Division of Biostatistics, University of Texas School of Public Health, Houston, TX 77025, USA; 2 Department of Breast Medical Oncology, University of Texas MD Anderson Cancer Center, Houston, TX 77030, USA; 3 Department of Biostatistics, University of Texas MD Anderson Cancer Center, Houston, TX 77030, USA; 4 Department of Biostatistics, Columbia University, Mailman School of Public Health, New York, NY 10032, USA

## Abstract

We are now well into the sequencing era of genetic analysis, and methods to investigate rare variants associated with disease remain in high demand. Currently, the more common rare variant analysis methods are burden tests and variance component tests. This report introduces a burden test known as the modified replication based sum statistic and evaluates its performance, and the performance of other common burden and variance component tests under the setting of a small sample size (103 total cases and controls) using the Genetic Analysis Workshop 18 simulated data with complete knowledge of the simulation model. Specifically we look at the variable threshold sum statistic, replication-based sum statistics, the C-alpha, and sequence kernel association test. Using minor allele frequency thresholds of less than 0.05, we find that the modified replication based sum statistic is competitive with all methods and that using 103 individuals leads to all methods being vastly underpowered. Much larger sample sizes are needed to confidently find truly associated genes.

## Background

We are now well into the sequencing era of genetic analysis, and methods to investigate rare variants associated with disease remain in high demand. The typical methods for detecting single variants associated with disease are not suited for the rare variants, owing to the low minor allele frequency (MAF) of rare variants. Most rare variant analysis methods fall into two categories, burden tests [1] and variance component tests [2]. Both of these classes of tests find association between rare variants and disease by pooling rare variants in a defined region in some sense. Whereas burden tests pool the change in risk (positive or negative) caused by total rare variants in a region, variance component methods compare the distribution of rare variant counts with that expected under the null.

We begin by briefly summarizing some of the more common and more recent methods for rare variant analyses and give their primary references. We consider the following burden tests: the variable threshold sum statistic (VT) [3] and the replication-based weighted sum statistic (RBS) [4] and the following variance component tests: C-alpha [5] and the sequence kernel association test (SKAT) for rare variants [2]. The variable threshold sum statistic computes a score for each region or gene based on a weighted sum of variant counts. The weights are defined by a combination of variant importance score, or frequency weight, and allow the minor allele threshold to vary according to locus to define the rare variant [3]. The VT method assumes the variants in each gene affect risk in the same direction. RBS constructs a weighted sum of rare variant scores, defining weights as a function of the minor allele being more frequent in the cases or controls and constructing the appropriate weighted sum score of those variants overrepresented in the controls (S_+_) or cases (S_-_) to accommodate both protective or deleterious rare variants within the same gene [4]. Then the final statistic to assess the association of rare variants to disease can be constructed in one of two ways. Take the maximum of the weighted sum of the variants more frequent in the cases and the weighted sum in the controls (S_max _= max[S_-_,S_+_]) or sum the weighted sums (S_comb _= S_- _+ S_+_) [4]. In this paper, we propose an additional RBS that combines S_- _and S_+ _based on the data. The C-alpha statistic is a variance component method that compares the distribution of the rare variants in the sample with that expected under the null, assuming a binomial distribution of each rare variant [5]. And finally, SKAT uses a linear model type approach to construct an aggregate weighted score test statistic for variants in a given region of interest [2]. Both C-alpha and SKAT can accommodate both risk and protective variants in the same gene.

Additionally, a major issue faced with researchers investigating rare variants, beyond the low MAF, is sample size considerations. Although some consortia exist, providing larger sample sizes for rare variant analyses, many researchers following up genome-wide association studies (GWAS) do not have the resources to sequence the full GWAS sample, leading to sample sizes on the order of only a few hundred for rare variant analysis. One recent study used 40 individuals in total [6].

As with all methods, larger samples give more power, and limited sample size limits the power. With rare variants, this is compounded because only a few individuals in the sample will exhibit a rare variant. For smaller samples, the MAF of the rare variant must be larger to even have a chance of appearing in the sample. But would a rare variant analysis still be useful for the rare variants that do appear in the sample? And which method would be effective in detecting the rare variants with smaller sample size? In addition to examining our new method, we investigate these questions using the simulated data of the Genetic Analysis Workshop 18 (GAW18) (see [7] for full details).

## Methods

In our study, we propose a modified RBS. We compare its performance and that of the burden and variance component tests mentioned earlier. The details of our modified RBS follow.

### The modified replication-based weighted sum statistic: S_tau_

We follow the notation from [4]. Let $n_{k_{+}}^{k^{\prime }}$ denote the number of variants in group (k',k) where k' denotes copies of the minor allele that appear in the cases and k denotes copies of the minor alleles that appear in the controls, and k' > k. Let $n_{k-}^{k^{\prime }}$ be defined as a similar count, except k' < k. We define S_+ _and S_- _as in [4], which are the two statistics for variants with k' > k and k' < k, respectively. Then we can define S_τ _as the weighted sum of weighted sums: $S_{tau}=\tau S_{+}+\left(1-\tau \right)S_{-}$, where $\tau =\frac{\underset{k^{\prime }>k}{\sum }n_{k_{+}}^{k^{\prime }}}{\underset{k^{\prime }>k}{\sum }n_{k_{+}}^{k^{\prime }}+\underset{k^{\prime }<k}{\sum }n_{k_{-}}^{k^{\prime }}}$. Defining τ in this way combines the concepts touched on in the S_max _and S_comb_. S_max _models the scenario of extremes, either all the rare variants are risk or all are protective, while the S_comb _assumes equal risk and protective rare variants. S_tau _allows the data to weight the impact of risk and protective variants according to the data, modeling unequal protective and risk variants in the combined statistic.

### Data preparation and phenotype definition

Because the methods detailed are designed for case-control data, we focus on the unrelated individuals. Using all of the longitudinal data, we define as a case any individual who became hypertensive over the course of observation, and we define as a control any individual who did not exhibit signs of hypertension. Across the 200 replicates, we have an average of 48 controls and 55 cases for a total of 103 unrelated individuals. (Of the total 159 unrelated individuals, 103 had all the information needed for this analysis.) Because we are comparing the performance of methods on the same data, we focused on genes on chromosome 3 that were used in the simulation model. We used NCBI dbSNP to identify all the single-nucleotide polymorphisms (SNPs) belonging to each gene on chromosome 3 involved in the simulation model.

We evaluated type 1 error, based on a resampling approach, specifically, we simulated a dichotomous phenotype to be independent of the genotype following the method in [8]. We simulate a Bernoulli random variable with event probability 0.5. If the variable = 1, we change the original phenotype to the alternate group, and if the variable =0, we keep the original phenotype status.

### Data analysis

For the burden tests, we computed a burden statistic for each gene. We computed S_max_, S_comb_, S_tau_, C-alpha, and VT for chromosome 3 for each replicate using the simulated phenotype. We also used the freely available R-package for computing SKAT using the default values with the small sample size option [2]. For each replicate, we recorded whether each method declared one of the solution genes as significant and reported the power for each method as the proportion of the 200 replicates where each method identified the gene as associated with the disease. We focused our analysis on rarer SNPs with MAFs less than 5%.

## Results

**Figure 1:**
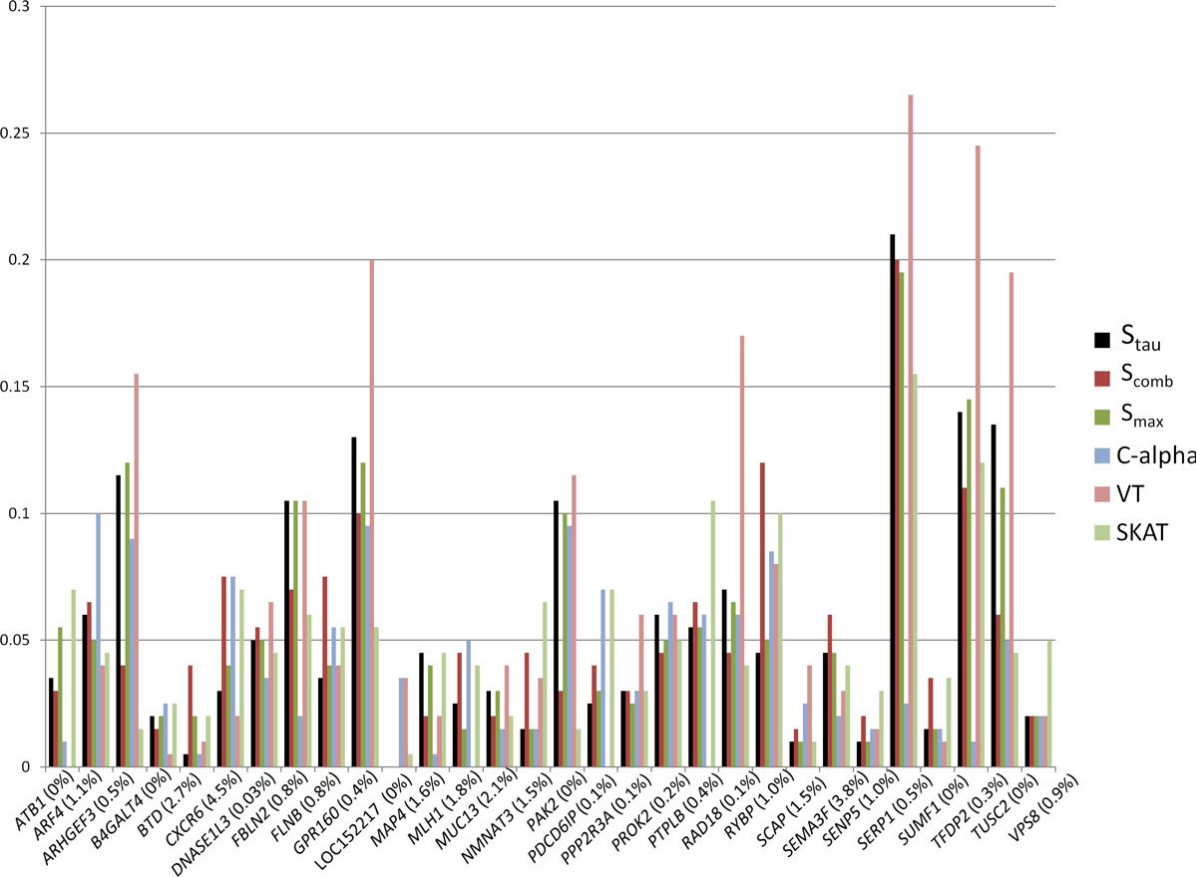
**Power for analysis with minor allele frequency (MAF) less than 0.05**. This figure shows the power of each method to detect each gene as causal for the 200 replicates of the simulation data when including only rarer variants with empirical MAF less than 0.05. The methods perform similarly within each gene. Especially note the performance of the modified replication based sum statistic S**_tau _**(solid black bar) relative to its predecessors S**_comb _**and S**_max_**. For these data, all 3 methods are very competitive. Below the horizontal axis is the percent signal for each gene (percent of causal rare variants divided by the total number of rare variants multiplied by 100).

The signal strength for each gene on chromosome 3 ranged from 0% to 4.5% (the number of causal rare variants divided by the total rare variants). The actual raw number of causal variants per gene ranged from 0 to 9, and the number of total variants per gene ranged from 5 to 2531. Thus, the total number of rare variants (denominator) controlled the signal to noise ratio (see Figure 1, horizontal axis). To compare the performance of each method on the nongenetic dichotomous phenotype, the type 1 error was controlled at the 0.05 level. Across all 200 simulated replicates, the average proportion of significance for all genes for each method was 0.05.

The modified replication based sum statistic, S_tau _performs competitively, especially compared with the other replication based statistics, S_max _and S_comb_, within 5% power of each other. In fact, most of the 6 methods performed similarly for each gene, (within 5%). Power exceeded 10% for only 9 of the 30 causal genes: *ARHGEF3, FBLN2, GPR160, PAK2, PTPLB *(SKAT only), *RAD18 *(VT only), *RYBP *(VT only) *SERP1, TFDP2*, and *TUSC2 *(Figure 1). Additionally, for some genes (*GRP160, SERP1, TFDP2, TUSC2*), the VT method is substantially more powerful than the other methods, but for others (e.g., *SEMA3F*), it is less powerful. Regardless of the variance in power across methods across genes, when averaging each method's power across all genes, the averages were close to 0.05 (Table 1).

**Table 1:** Mean power for each method across all genes

Method	Hypertension	Nongenetic
S_tau_	0.056	0.048
S_comb_	0.083	0.049
S_max_	0.055	0.046
C-alpha	0.043	0.050
VT	0.069	0.050
SKAT	0.051	0.055

This table reports the mean power for each of the 6 methods investigated for those variants with minor allele frequencies less than 0.05. We report the power for the simulated hypertension phenotype as well as for a nongenetic phenotype for type 1 error.

*SKAT*, sequence kernel association test; *VT, v*ariable threshold.

## Discussion

The new method we introduced in this paper, S_tau, _performs competitively with other popular methods for rare variant analysis. It does not exhibit false positives much higher than the other methods, nor does it stand alone in its detecting or failing to detect the causal variants. However, given the small sample size, it is difficult to identify any new advantages or disadvantages of this modified replication-based method.

In fact, all methods investigated here are underpowered for this case-control study with a sample size of 103 individuals. These methods fail to detect the causal rare variants most of the time (Figure 1). Even more complex, there is not an easily identified pattern or rationale to when these methods do well for this sample size. For instance, all methods exhibited poor power (<0.05) to detect *MAP4*, which was simulated to be one of the top 15 genes with the largest effect sizes for any gene on chromosome 3. And yet *TUSC2 *has only one causal variant according to the simulation model parameters, whose minor allele was not present in our sample. As a gene, it is much smaller with only 19 rare variants compared with *MAP4 *with more than 400 variants. Yet it only has a few variants analyzed, and 2 of them are in linkage disequilibrium (LD) (R^2 ^>0.25) with a causal variant in *MAP4 *that has a large effect size for both diastolic blood pressure (DBP) and systolic blood pressure (SBP). Because this is a gene detected by all methods with power greater than 0.2, the power could be driven through the LD with the large effect size combined with the small number of total variants included in the weighted sum. Beyond gene size and LD, using a dichotomous hypertension variable rather than the continuous trait could have cost power and added complexity to determine the efficacies of these methods with such a small sample size versus using SBP or DBP as a continuous trait.

## Conclusions

The main conclusion of this paper is that for all methods, 103 individuals are not enough for a rare variant analysis of a complex qualitative disease such as hypertension. Perhaps power can be increased using blood pressure as a continuous trait rather than treating hypertension status as a qualitative trait. A secondary conclusion from this report is that the new method S_tau _performs similar to its predecessors (S_comb _and S_max_). It is worth further investigation to more clearly determine any advantages for using S_tau_.

## Competing interests

The authors declare that they have no competing interests.

## Authors' contributions

MDS designed the overall study, conceived of S_tau_, performed some analyses, and wrote the manuscript. TK refined S_tau_; wrote computer code to compute S_tau_, S_max_, S_comb_, and a script to perform all analyses; and provided editorial input on the manuscript. JN wrote computer code to compute VT and C-alpha and provided editorial input on the manuscript. RKY performed data cleaning and preliminary analyses and provided editorial input on the manuscript. SS consulted on the analysis and interpretation of results and provided editorial input on the manuscript II-L provided consultation for the computer code for S_tau_, S_max_, and S_comb_; analysis and interpretation of results; and editorial input on the manuscript. All authors read and approved the final manuscript.
